# Galactose-deficient IgA1 and nephritis-associated plasmin receptors as markers for IgA-dominant infection-related glomerulonephritis: A case report

**DOI:** 10.1097/MD.0000000000024460

**Published:** 2021-02-05

**Authors:** Wei Han, Tomo Suzuki, Shiika Watanabe, Mayumi Nakata, Daisuke Ichikawa, Junki Koike, Takashi Oda, Hitoshi Suzuki, Yusuke Suzuki, Yugo Shibagaki

**Affiliations:** a Division of Nephrology and Hypertension, Department of Internal Medicine, St Marianna University School of Medicine, Kawasaki; b Department of Nephrology, Kameda Medical Center, Chiba; c Department of Diagnostic Pathology, St Marianna University School of Medicine, Kawasaki; d Department of Nephrology, Tokyo Medical University Hachioji Medical Center; e Department of Nephrology, Juntendo University Faculty of Medicine, Tokyo, Japan.

**Keywords:** galactose-deficient IgA1, IgA nephropathy, infection-related glomerulonephritis, nephritis-associated plasmin receptor

## Abstract

**Rational:**

Immunoglobulin A (IgA) nephropathy is a common heterogeneous kidney disease. One of the causes of secondary immunoglobulin A nephropathy is infection-related glomerulonephritis (IRGN), however, its accurate diagnosis is difficult.

**Patient concerns:**

We report a rare case of an 82-year-old male presenting rapidly progressive glomerulonephritis. Assessment of a kidney biopsy by light microscopy revealed endocapillary glomerulonephritis with subendothelial deposits, such as wire loop lesions and cellular crescents. Immunofluorescence demonstrated strong staining for IgA and C3 along the glomerular capillary. Additional tests included positive staining for nephritis-associated plasmin receptor and positive plasmin activity in the glomeruli. Moreover, IgA and galactose-deficient IgA1 (Gd-IgA1) staining merged using immunofluorescence, followed by confirmation of high serum levels of Gd-IgA1 (9.3 μg/mL) by ELISA was observed.

**Diagnosis:**

The diagnosis of IgA-dominant IRGN was made.

**Interventions and outcomes:**

We have initiated treatment with intravenous methylprednisolone 500 mg/day for 3 days, followed by oral prednisolone 25 mg/d as rapidly progressive glomerulonephritis. However immunosuppressive therapy was halted because of a poor response, and hemodialysis was initiated.

**Lessons:**

This is a case of IgA-dominant IRGN patient exhibiting positive glomerular staining for nephritis-associated plasmin receptor accompanied with high titers of serum Gd-IgA1. Our observations suggest that serum and kidney tissue of Gd-IgA1 may be useful for the diagnosis of IgA-dominant IRGN.

## 1 Introduction

IgA nephropathy (IgAN) is the most common form of glomerulonephritis worldwide.^[1]^ Galactose-deficient IgA1 (Gd-IgA1) is known to deposit specifically in the glomeruli of patients with IgAN.^[2]^ However, histological features of IgAN is various as known in the Oxford Classification, which includes findings on light microscopy such as, mesangial hypercellularity, endocapillary hypercellularity, segmental glomerulosclerosis, tubular atrophy/interstitial fibrosis, and crescents. In addition, it is well documented that secondary IgAN is induced by various conditions, such as gastrointestinal disease, liver disorder, autoimmune disorders, and infection.^[3]^ Among secondary IgAN, infection-related glomerulonephritis (IRGN) associated especially with staphylococcus species, induces glomerulonephritis due to the formation of IgA-dominant immunocomplex deposits. However, it remains unclear whether there is an association between IgA-dominant IRGN and Gd-IgA1.

Herein, we report a case of a patient with IgA-dominant IRGN exhibiting positive staining for nephritis-associated plasmin receptor (NAPlr) and plasmin activity. A high titer of serum Gd-IgA1 as well as Gd-IgA1-positive staining of the kidney tissue was observed, and we speculate that the resultant glomerulonephritis is due to overproduction of Gd-IgA1 induced by infection.

## 2 Case report

An 82-year-old male experiencing bilateral edema at the lower extremities after a fall was referred for evaluation. Upon examination, rapid renal deterioration was noted as serum creatinine rose from 2.32 to 3.12 mg/dL over the course of 3 weeks, after which he was subsequently referred to our hospital and admitted with suspected rapidly progressive glomerulonephritis. Medical history included hypertension and hyperuricemia during a previous hospitalization. The patient consumed high levels of alcohol; daily intake of 300 to 500 ml Japanese sake. Additionally, he had been taking amlodipine 5 mg/day and febuxostat 10 mg/day.

Upon admission, physical examination and laboratory findings determined the following profiles: height at 155 cm, weight 63.4 kg, and BMI 26.2 kg/m^2^. Blood pressure was slightly elevated at 151/67 mmHg and serum creatinine levels had increased to 5.18 mg/dL. A complete blood count indicated: white blood cell was 5400 / ml (neutrophil 60%), hemoglobin was 11.2 g/dL and platelet count was 8,6000/ml. Chemical analysis showed total protein at 5.1 mg/dl, albumin at 1.9 g/dl, LDL-cholesterol at 143 mg/dL, and triglyceride at 123 mg/dL. Urinalysis revealed 3+ proteinuria and 3+ hematuria. In urine sediment, red blood cell count was greater than 100 per high-power field. The spot urine protein level was 4.5 g/g creatinine. Serological examination revealed a high level of IgA at 557 mg/dL. Other immunoglobulins measured were normal; IgG 1234 mg/dL and IgM 55 mg/dL. Testing for anti-nuclear antibody (ANA) was positive at 1:40 with a speckled pattern. However, anti-double-stranded DNA antibody gave negative results. In addition, myeloperoxidase-antineutrophil cytoplasmic antibodies (ANCA) and proteinase 3-ANCA were not detected. At time of hospitalization complement levels were unaffected: C3 66 mg/dL (normal range 65 to 135) and C4 25 mg/dL. After 6 days, C3 slightly decreased to 64 mg/dL in spite of the acute inflammatory phase, however, then increased again and remained within normal range.

**Figure 1:**
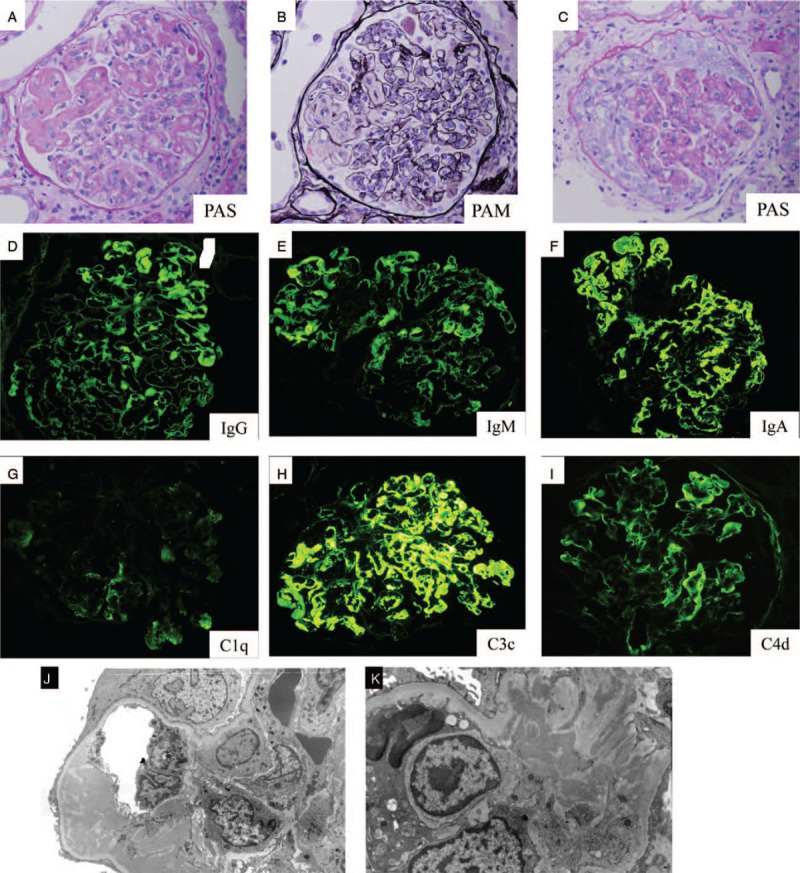
Light microscopy shows staining with periodic acid-Schiff (PAS) and periodic acid–methenamine silver stain (PAM). Original magnification × 200 (A, B) Glomerulus exhibits massive subendothelial deposits, such as wire loop lesion and endocapillary proliferation. (C) Glomerulus displays cellular crescents and endocapillary proliferation. Immunofluorescence of kidney biopsy shows staining with (D) IgG, (E) IgM, (F) IgA, (G) C1q, (H) C3c, (I) C4d. Original magnification × 200. Electron microscopy shows mesangial and endothelial unorganized electron dense deposit, in (J) low and (K) high magnification.

A kidney biopsy was performed and the specimen was shown to contain 17 glomeruli, with 7 global sclerosis. Light microscopy showed various characteristics, such as subendothelial deposits including wire loop lesions, endocapillary proliferation, and cellular crescents in the glomeruli (Fig. 1 A-C). immunofluorescence (IF) revealed strong positive staining for IgA and C3 along the glomerular capillary. In contrast, IgG, IgM, C1q, and C4d were weakly stained, in particular C1q (Fig. 1 D-I). Non-structured massive electron dense deposits were observed in the subendothelial and mesangial area using electron microscopy (Fig. 1 J, K)

**Figure 2:**
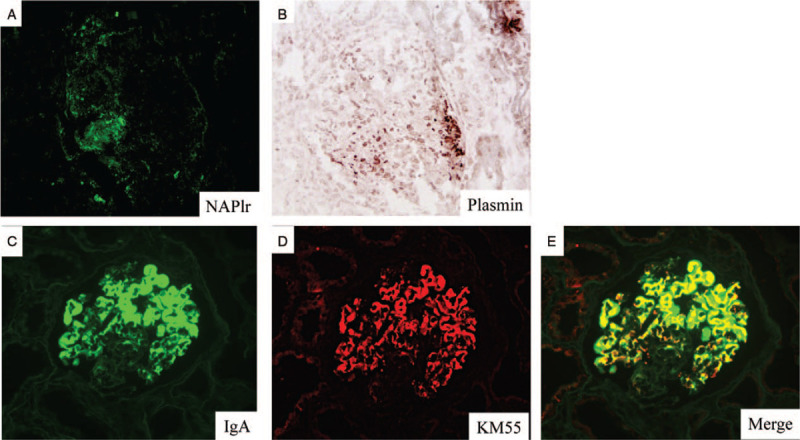
Immunostaining of kidney biopsy specimen. Original magnification × 200. (A) Glomerulus staining for nephritis-associated plasmin receptor (NAPlr) by immunofluorescence and (B) *in situ* zymography to determine plasmin activity using plasmin sensitive synthetic substrate. (C, D, E) Glomerulus is positively stained for IgA and Gd-IgA1 and Merge on immunofluorescence.

Treatment was initiated with intravenous methylprednisolone 500 mg/d as a steroid pulse therapy for 3 days, followed by oral prednisolone 25 mg/d as it was considered rapidly progressive glomerulonephritis. As strong positive staining of IgA and C3 was observed compared to IgG and C1q, we suspected IRGN and therefore included further testing of NAPlr and plasmin activity to determine infection. Whilst presence of infection was not clear from the tests performed on blood culture, head non-contrast computed tomography (CT), contrast CT from chest to abdomen and lumbar magnetic resonance imaging; positive NAPlr and plasmin activity was detected in the glomeruli (Fig. 2 A, B). Immunosuppressive therapy was halted because of a poor response to this treatment by the patient, and hemodialysis was initiated. His Renal function did not recover and hemodialysis was continued. Two months after dialysis initiation, he was transferred to another hospital for maintenance hemodialysis treatment.

Further investigation confirmed that IgA and Gd-IgA1 staining merged in IF (Fig. 2 C, D, E). In addition, high levels of serum Gd-IgA1 (9.3 μg/mL) were detected by ELISA. As this result is high compared to that seen in other kidney diseases,^[2]^ the final diagnosis of IgA-dominant IRGN was concluded.

## 3 Discussion

This report presents a case of a patient exhibiting IgA-dominant IRGN with positive staining for NAPlr and plasmin activity in the glomeruli of the kidney biopsy tissue. Moreover, Gd-IgA1-positive staining of the kidney tissue corroborated the results of the high serum Gd-IgA1 titers.

We have previously diagnosed IRGN based on the diagnostic criteria together with the evidence of infection. According to the IRGN diagnostic criteria reported by Nasr et al, our case met 3 out of 5 criteria which include depressed serum complement, endocapillary and exudative glomerulonephritis, and C3 co-dominant glomerular immunofluorescence staining.^[5]^

NAPlr is a nephritogenic protein isolated from group A streptococcus (GAS). Recent reports also describe positive NAPlr staining in IRGN induced by bacteria *Streptococcus pneumoniae*,^[6]^*Aggregatibacter actinomycetemcomitans* (a component of the Gram-negative oral flora),^[7]^ and *Mycoplasma pneumoniae*.^[8]^ Therefore, NAPlr and plasmin activity have been proposed as the general biomarkers of bacterial IRGN.^[9]^ On this basis, we diagnosed our case as IRGN, however, infection was not detected clinically. Whilst no cases of IgA-dominant IRGN with positive glomerular staining for NAPlr have been published to date, Kikuchi *et al* have reported a case of Henoch-Schonlein purpura (IgA vasculitis) with positive staining for NAPlr and plasmin activity in kidney glomeruli.^[10]^ Interleukin-6 (IL-6), a cytokine generally produced during infection, is reported to increase IgA1 synthesis and can also accentuate the degree of galactose deficiency of IgA1.^[11]^ Thus, we suggest that the present case shows glomerulonephritis due to overproduction of Gd-IgA1 induced by infection. This is strongly supported by the finding of this case showing positive staining observed for both NAPlr and Gd-IgA1 in glomeruli of the kidney tissue.

Various IF staining patterns of IgA-dominant IRGN are described, with Nasr et al reporting 8 out of 48 IgA-dominant IRGN cases positive for C1q.^[4]^ Moreover, Satoskar *et al* reported 4 out of 8 cases of IgA-dominant staphylococcus infection-associated glomerulonephritis were IgG positive.^[12]^ Due to the variety of potential immunostaining patterns it is difficult to diagnose IgA-dominant IRGN.

Gd-IgA1 is detected in IgA vasculitis as well as in IgAN.^[13]^ Furthermore, Wang et al recently reported that secondary IgAN shared similar Gd-IgA1 with primary IgAN.^[14]^ We believe the Gd-IgA1 was triggered by infection, since the secondary IgAN observed (IgA of lupus nephritis, hepatic IgAN, and Gd-IgA1) did not merge.^[9]^ Additionally, as observed in our case, the Gd-IgA1 of staphylococcus associated glomerulonephritis showed weak to strong positive results.^[15]^ Therefore, we suggest the therapeutic usefulness of Gd-IgA to diagnosis IRGN in kidney tissues, particularly when the case presents with Full house (IgG, IgA, IgM, C1q, C3, positive immunostaining) pattern.

In conclusion, this case report describes the detection of Gd-IgA1 positive kidney tissue which is mirrored with a high serum titer of Gd-IgA1, in an IgA-dominant IRGN patient displaying a full house IF pattern. Serum and kidney tissue Gd-IgA1 may be useful markers for the diagnosis of IgA-dominant IRGN.

## Author contributions

HW and TS wrote the first draft. TS, SW, MN, DI, and JK contributed to the patient's care and histological interpretation. TO examined NAPlr and plasmin staining. HS and YSu examined serum ELISA and staining of Gd-IgA1. TO, YSu, and YSh contributed by reviewing and revising the manuscript.

**Conceptualization:** Tomo Suzuki, Takashi Oda, Yusuke Suzuki.

**Data curation:** Shiika Watanabe, Mayumi Nakata.

**Supervision:** Tomo Suzuki.

**Writing – original draft:** Wei Han, Tomo Suzuki.

**Writing – review & editing:** Tomo Suzuki, Daisuke Ichikawa, Junki Koike, Takashi Oda, Hitoshi Suzuki, Yusuke Suzuki, Yugo Shibagaki.
